# Phenotypical and Functional Analysis of Intraepithelial Lymphocytes from Small Intestine of Mice in Oral Tolerance

**DOI:** 10.1155/2012/208054

**Published:** 2012-02-07

**Authors:** Maristela Ruberti, Luis Gustavo Romani Fernandes, Patricia Ucelli Simioni, Dirce Lima Gabriel, Áureo Tatsumi Yamada, Wirla Maria da Silva Cunha Tamashiro

**Affiliations:** ^1^Department of Genetics, Evolution and Bioagents, Institute of Biology, University of Campinas (UNICAMP), Rua Monteiro Lobato of 255, CP 6109, 13083-970 Campinas, SP, Brazil; ^2^Department of Histology and Embryology, Institute of Biology, University of Campinas (UNICAMP), CP 6109, 13083-970 Campinas, SP, Brazil

## Abstract

In this work, we evaluated the effects of administration of OVA on phenotype and function of intraepithelial lymphocytes (IELs) from small intestine of transgenic (TGN) DO11.10 and wild-type BALB/c mice. While the small intestines from BALB/c presented a well preserved structure, those from TGN showed an inflamed aspect. The ingestion of OVA induced a reduction in the number of IELs in small intestines of TGN, but it did not change the frequencies of CD8^+^ and CD4^+^ T-cell subsets. Administration of OVA via oral + ip increased the frequency of CD103^+^ cells in CD4^+^ T-cell subset in IELs of both BALB/c and TGN mice and elevated its expression in CD8**β** ^+^ T-cell subset in IELs of TGN. The frequency of Foxp3^+^ cells increased in all subsets in IELs of BALB/c treated with OVA; in IELs of TGN, it increased only in CD25^+^ subset. IELs from BALB/c tolerant mice had lower expression of all cytokines studied, whereas those from TGN showed high expression of inflammatory cytokines, especially of IFN-**γ**, TGF-**β**, and TNF-**α**. Overall, our results suggest that the inability of TGN to become tolerant may be related to disorganization and altered proportions of inflammatory/regulatory T cells in its intestinal mucosa.

## 1. Introduction

Oral tolerance has long been recognized as a physiological mechanism of immune unresponsiveness to dietary antigens (Ags) and indigenous bacterial Ags that maintains tissue integrity by preventing harmful delayed type hypersensitivity responses in the intestine [1]. Three mechanisms were postulated to mediate oral tolerance: clonal deletion, anergy, and active immune suppression. Lower doses of Ag have favored active suppression, and this mechanism is mediated through regulatory cytokines such as TGF-*β*, IL-10, and IL-4 [2]. T cells producing these regulatory cytokines may downregulate autoreactive T cells in an antigen nonspecific way [2, 3], whereas higher doses favor anergy and T-cell depletion [3]. More recent results show that an activation of T cells is necessary before establishing oral tolerance [4], while enhanced antibacterial immunity can be achieved with concomitant generation of oral tolerance followed by oral administration of soluble antigen such as ovalbumin (OVA) [5]. 

The immunological consequences of oral administration of antigen ultimately depend on how antigen is taken up and presented to T cells by dendritic cells [6]. Despite of the initiation of oral tolerance remains to be cleared, it seems to involve the active participation of the all gut-associated lymphoid tissue (GALT) [7]. Intraepithelial lymphocytes (IELs) play an important role in the maintenance of mucosal homeostasis by regulating mucosal innate and acquired immunity [8]. The populations of IELs exhibit unique characteristics, perform functions not fully elucidated, and differ widely from their systemic counterparts [9]. IELs in mice and humans include large numbers of cells expressing T-cells receptor (TCRs) *αβ* and *γδ* [9]. The majority of IELs are CD8^+^ cells that express a CD8*αα* homodimer. Among IELs subsets, it can still be found some CD4^−^CD8^−^double negative cells, CD4^+^CD8^+^ double positive cells and a few CD4^+^CD8*αα* ^+^ cells [8, 10]. Nearly all CD8^+^ IELs expressed CD69 and had lytic activity [11]. 

 Preliminary results of our group have shown that DO11.10 mice, that bear transgenic anti-OVA TCR, are not susceptible to oral tolerance with OVA [12]. In the present work, we investigated the immune response that takes place in intestinal mucosa during the consumption of OVA in both BALB/c and DO11.10 mice. Since the majority of studies with oral tolerance induction and mucosal immune response have only evaluated the role of CD4^+^ *αβ* T cells [13–15], we analyzed all subsets of IELs of these mice in the context of oral tolerance and immune response to OVA.

## 2. Material and Methods

### 2.1. Animals

Breeder pairs of TCR OVA-specific transgenic mice (clone DO11.10) [16] and BALB/c mice were supplied by CEMIB (Centro Multinstitucional de Investigacões Biológicas), UNICAMP. Mice were maintained under specific pathogen-free condition and were provided with autoclaved food and water. The study was approved by the Ethics Commitee for Animal Experimentation of University of Campinas (Protocol no. 736-2). 

### 2.2. Tolerance Induction and Immunizations

Oral tolerance to OVA was induced in 8 weeks old mice as described elsewhere [12]. Briefly, Mice were fed with 4 mg/mL OVA solution (Rhoster Indústria e Comércio, Ltda., Vargem Grande Paulista, SP, Brazil) for seven consecutive days. The mice in the control group received protein-free water. Seven days after the interruption of oral treatment, half of this group of mice was challenged with of 10 *μ*g OVA (Sigma Chemical Co., St. Louis,MO, USA) plus 1 mg Al(OH)_3_ by intraperitoneal (ip) route. After 14 days, mice were boosted with 10 *μ*g OVA in saline solution via ip. In control group, half of the animals were also challenged with the ip doses of OVA. Seven days after the last ip dose, all mice were bled for serum separation, and then euthanized in a CO_2_ chamber. 

### 2.3. Cell Isolation and Purification

Intraepithelial lymphocytes were isolated from the small intestine of BALB/c and DO11.10 mice, according to Montufar-Solis and Klein [17]. Briefly, small intestine tissues were removed, and Peyer's patches were dissected out. Tissues were flushed of fecal material, opened longitudinally, and cut into 3 to 4 mm pieces in RPMI 1640 (Sigma-Aldrich) supplemented with FCS (10% v/v) (Nutricell, Campinas, SP, Brazil), gentamicin 20 *μ*g/mL (GE Healthcare Biosciences, Pittsburgh, PA, USA). Tissue fragments were rinsed several times in Ca2^−^/Mg2^−^ free PBS, transferred to Ca2^−^/Mg2^−^ free PBS containing 5 mM EDTA (Sigma-Aldrich) and 2 mM DTT (Calbiochem; Cleveland's reagent; Merck KGaA, Darmstadt, Germany) and shaken (bath Dubnoff-Marconi, Piracicaba, SP, Brazil) at 37°C for 30 min. Cell suspensions were filtered through 20-mL syringe barrels containing wetted nylon wool, centrifuged, suspended in 3 mL of 40% isotonic Percoll (Amersham and Sigma-Aldrich), layered on top of 70% isotonic Percoll (4 mL), and centrifuged for 30 min at 400 xg. IELs were recovered from the Percoll interface and washed by centrifugation in supplemented RPMI-1640, viability and cell numbers were measured by exclusion of Trypan blue dye and counting in a hemocytometer. IELs from BALB/c mice were further purified by immunomagnetic separation, using beads conjugated with mAb anti-CD90 (Thy-1.2) on MS columns, as recommended by the manufacturer (Midi Macs, Miltenyi Biotec, Bergisch-GladBach, Germany).

### 2.4. Phenotypic Analysis by Flow Cytometry

Single-cell suspensions from small intestine were suspending in PBS/0,01% BSA (Sigma) w/v supplemented with 0,1% sodium azide. Cells were first incubated with anti-CD16/32 (culture supernatants of clone 2.4G2) for 45 min to block Fc-mediated antibody binding. Then, cells were incubated with relevant mAb for 30 min at 4°C, washed with PBS/2% FCS, and fixed 9 with PBS/1% formaldehyde (Merck Darmstadt, Germany). Three- or four-color flow cytometry acquisition was performed on FACSAria (BD Bioscience, San Jose, CA, USA). A total of 30.000 events were acquired in each analysis. The following antibodies purchased from BDPharMingen were used for flow cytometry: anti-CD3 (clone 145-2C11)-PerCP Cy5.5 or FITC; anti-CD4 (clone GK1.5)-FITC, PE or PE-Cy7; anti-CD4 (clone RM4-5)-PE-Cy7; anti-CD8*α* (clone 53-6.7)-FITC or PE; anti-CD8*β* (clone 53-5.8)-FITC; anti-TCR*β* (clone H57-597)-PE; anti-TCR*γδ* (clone GL3)-FITC or PE; anti-CD152 (CTLA-4) (clone UC10-4F10-11)-PE; anti-CD25 (clone 7D4)-FITC or PE; anti-CD103 (*α* IEL) (clone M290)-PE; anti-OVA TCR (clone KJ1-26)-PE. Anti-Foxp 3 (clone FJK-16 s)-PE or FITC were purchased from eBioscience (San Diego, CA, USA). Data were analyzed by the software FCS express V3. Respective isotype controls were included for each cell surface stain to exclude nonspecific binding and to determine the optimal setting fluorescence quadrants (BD Bioscience, San Jose, CA, USA). Data were analyzed by the software FCS express V3 (De Novo Software, Los Angeles, CA, USA). 

### 2.5. Histological and Immunohistochemical Staining

For histological analysis, pieces from small intestine were fixed in 4% paraformaldehyde buffered solution (Sigma) and washed with PBS/1% Glycine (J.T. Baker, Mallinckrodt Baker, Phillipsburg, NJ, USA), and 5 *μ*m paraffin-embedded sections were stained with hematoxylin and eosin (Sigma). Slides were observed in an optical microscope (Eclipse E-800 Microscope, NIKON; Tokyo, Japan) and analyzed using the software Proplus image. For immunofluorescence analysis, sections of small intestine were dehydrated, frozen in OCT-embedding compound (Leica) on dry ice, and stored at −70°C. Tissue sections (5 *μ*m) were cut with a cryostat (Microm HM 505 E) and transferred to silane-coated microscope slides. Cryosections were brought to room temperature, fixed with acetone (Merck) for 10 min at 4°C, and blocked with PBS containing 1% of BSA (type V, INLAB, SP, Brazil) for 30 min. After washing with PBS, they were incubated with anti-CD3 FITC-labelled (clone 145-2C11 homemade) for 3 h, washed, and incubated with TRITC-phalloidin (Sigma) for 30 min. All incubations were made at room temperature. Vectashield-mounted slides (Vector Laboratories) were visualized by optical microscopy. 

### 2.6. Quantitative Real-Time Polymerase Chain Reaction (PCR)

Total RNA was extracted from mouse IELs using PureLink Micro-to-Midi Total RNA Purification System (Invitrogen, SP, Brazil,) according to the manufacturer's instructions. The cDNA was made using SuperScript III First-Strand Synthesis Supermix (Invitrogen) with random primers (Invitrogen) and analyzed for IL-2, IL-4, IL-10, IL-6, IL-17, IFN-*γ*, TGF-*β*, and TNF-*α* gene expression by real-time PCR assay using an 7500 Fast Real-Time PCR (Applied Biosystems, Foster City, CA) according to the manufacturer's instructions; 18S ribosomal RNA (rRNA) was used as an internal control. All mouse primer and probe sets used were predesigned TaqMan Gene Expression Assays (Applied Biosystems). PCRs were performed in four replicates with a 2x TaqMan Mastermix (Applied Biosystems). Relative expression of mRNA species was calculated using the comparative 2 threshold cycle (ΔCT) method [18]. 

### 2.7. Statical Analysis

The statistical analysis was performed using GraphPad Prism 4 (GraphPad Software, CA, USA). The statistical significance of differences between control and experimental groups were determined by one-way and two-way ANOVA, followed by multiple comparison Bonferroni's test. The results were expressed as mean ± SEM. Values were considered significant at *P* < 0.05. Supplemental data include two figures (see supplementary material available online at doi:10.1155/2012/208054). 

## 3. Results

### 3.1. Histological Analysis and Distribution of IELs in Small Intestines of Mice DO11.10 and BALB/c

**Figure 1:**
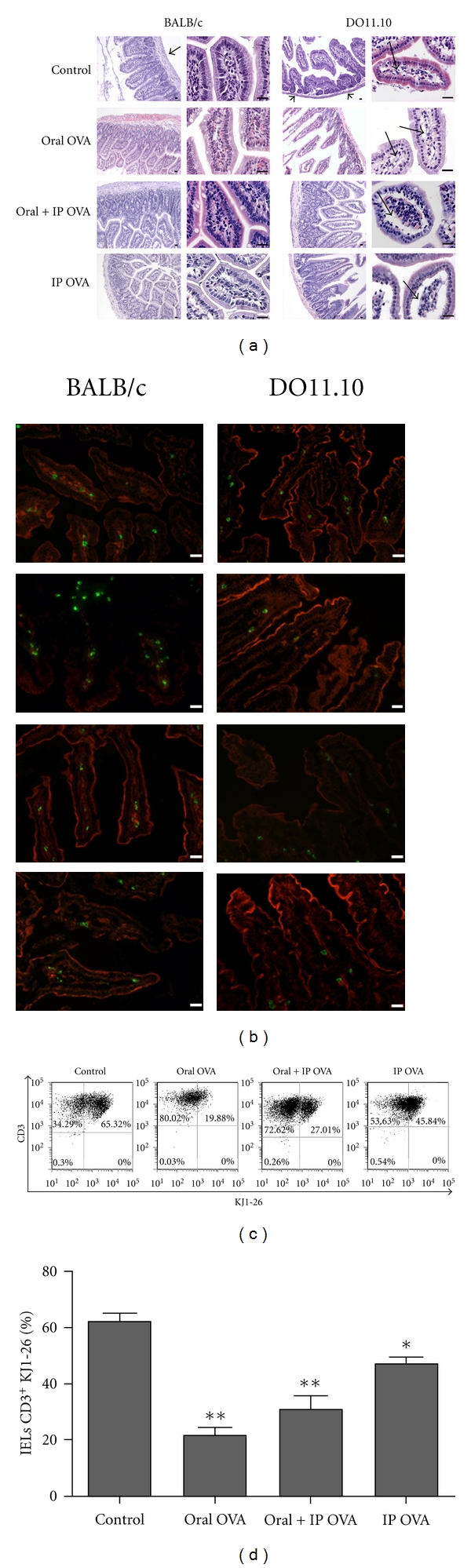
Histological analysis and incidence of T cells in small intestines (jejunum) of BALB/c and DO11.10 mice after treatments with OVA. Mice were fed with OVA solution for 7 days (oral OVA), fed with OVA and challenged by ip route (oral + ip OVA), immunized with OVA only by ip route (ip OVA), or non-treated (control). (a) Hematoxylin/Eosin-stained sections of small intestines in low and high magnification showing details of mucosa villi. Note the reduced thickness of the tunica muscular (arrow heads) in DO11.10 when compared with BALB/c, and loss of connective tissue and mild edema in the lamina propria (thin arrows) in Oral + ip OVA and ip OVA groups of DO11.10 mice. Bars = 50 *μ*m. (b) Immunofluorescence of frozen sections of jejunum counterstained with TRITC-phalloidin (red epithelial cells) showing reduced incidence of CD3 positive cells (green) in the mucosa of DO11.10 in comparison to BALB/c mice. Bars = 25 *μ*m; (c, d) Frequency of KJ1-26 positive cells amongst the intraepithelial lymphocytes freshly isolated from DO11.10 mice treated with OVA. The clonotype anti-OVA TCR cells (KJ1-26^+^ cells) decreased from 65% to less than 20% after oral and ip administration of OVA. Data represent mean ± SEM (*N* = 5) of three independent experiments.

As depicted in Figure 1(a), the small intestine histoarchitecture of both naïve DO11.10 and BALB/c strains were preserved; however, it was found reduced tunica muscular thickness of DO11.10 when compared with BALB/c mice. Discrete but well-defined histological changes were observed in the lamina propria (LP) of intestinal villi of the transgenic mice after feeding with OVA, mainly in those challenged with OVA by ip route, with a loose connective tissue rupture and mild edema of lamina propria of villous projections in DO11.10 mice. BALB/c mice treated with OVA did not present any of those alterations. The total number of IELs isolated from the small intestine of DO11.10 mice of all experimental groups was always lower than those from BALB/c and markedly dropped upon OVA treatments (Table 1). As illustrated in Figure 1(b), the incidence of CD3 positive cells decreased substantially in the villi of TGN mice but not in the BALB/c. Cytometry analyses of IELs isolated from TGN showed that the clonotype anti-OVA TCR cells (KJ1-26 positive cells) decreased significantly from 65% to less than 20% after oral and ip administration of OVA (Figures 1(c) and 1(d)).

### 3.2. Analysis of Subsets of IELs after the Induction of Tolerance or Immunization

**Figure 2:**
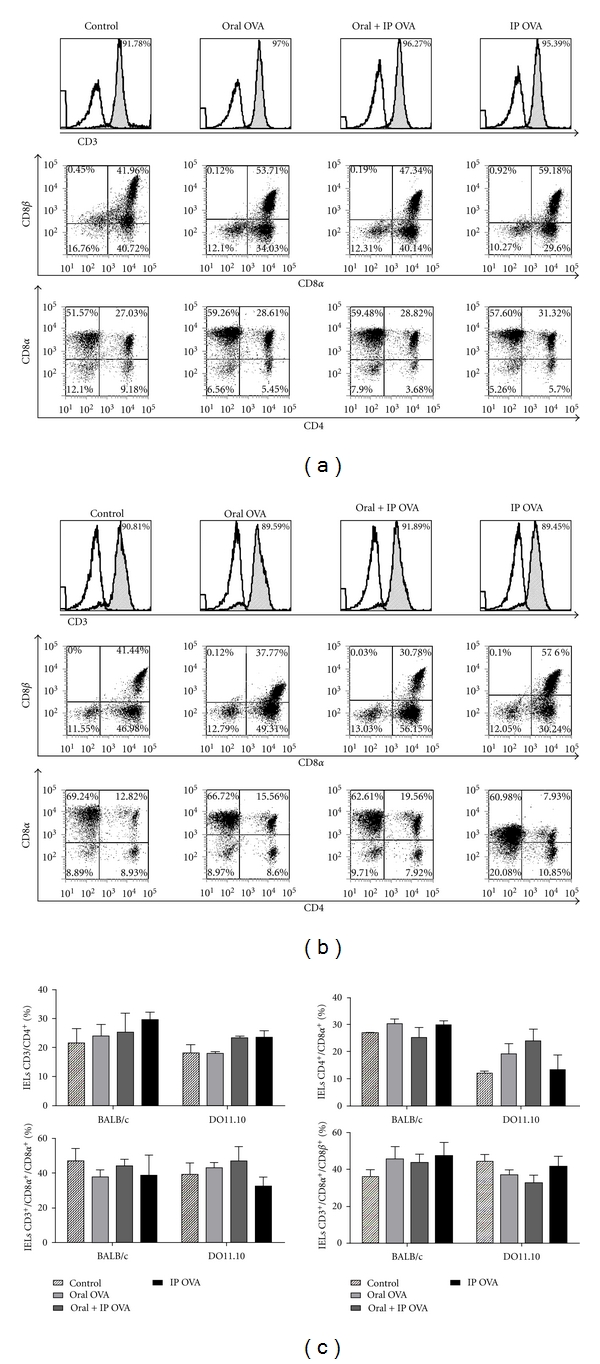
Effects of the treatments with OVA on CD4 and CD8 subsets of IELs. Freshly isolated IELs from BALB/c (a) and DO11.10 (b) mice were gated for CD3^+^ cells and analyzed for expression of CD8*α*, CD8*α*/CD8*β*, and CD4/CD8*α* by flow cytometry. No significant difference was found in the frequency of IELs of any subset in both strains of mice. In (c), data represent mean ± SEM (*N* = 5) in each group in three independent experiments.

IELs from BALB/c and D011.10 mice treated with OVA by oral and/or ip route were stained with anti-CD3, anti-CD8*α*, anti-CD8*β*, anti-CD4 and analyzed by three-color flow cytometry (Figure 2). As shown in Figure 2(a), the frequency of CD3^+^ cells in the small intestine of BALB/c and DO11.10 mice was not changed by different treatments with OVA. No significant alteration was observed in the frequency of IEL subsets (CD8*αα*, CD8*αβ*, CD4/CD8*α*, CD4) upon treatments with OVA (Figure 2(c)). 

**Figure 3:**
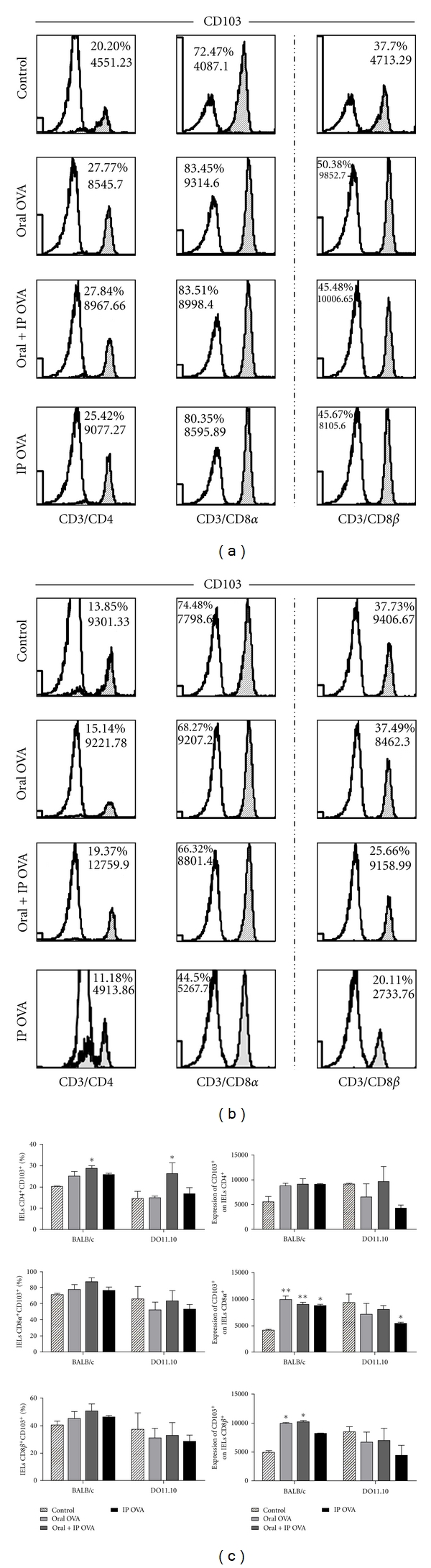
Effects of the treatments with OVA on the frequency of CD103^+^ cells in the small intestine. Freshly isolated IELs from BALB/c (a) and DO11.10 (b) mice were gated for CD3^+^/CD4^+^, CD3^+^/CD8*α* ^+^, and CD3^+^/CD8*β* ^+^ cells and analyzed for expression of CD103 by flow cytometry. Blank histograms indicate isotype control staining. CD3^+^/CD8*β* ^+^ subset illustrated at the right column is part of the CD3^+^/CD8*α* ^+^ population. (c) Data represent mean ± SEM (*N* = 5) in three independent experiments. Frequency of CD4^+^ cells were significantly more elevated in IELs isolated from mice BALB/c and DO11.10 treated with OVA by oral + ip routes. The expression of CD103 was markedly augmented in all subpopulations of IELs of OVA-treated BALB/c mice and was reduced in DO11.10 immunized by ip route.

The effects of administration of OVA on the distribution of phenotypic markers CD103 and CD25 were assessed in subsets CD4, CD8*α*, and CD8*β* of IELs isolated from BALB/c(Supplemental Figure  1, Panel A-D) and DO11.10 mice(Supplemental Figure  1, Panel (E-H)), as well as the frequencies of *αβ* ^+^ and *γδ* ^+^ T cells in these subsets (Supplemental Figure  2). Frequency and expression of CD103^+^ cells in subsets of IELs of BALB/c and DO11.10 mice are illustrated in histograms of (Figures 3(a) and 3(b), resp.). Significant increase of CD4^+^CD103^+^ subset of IELs was observed following treatments with OVA by oral + ip routes, in both BALB/c and DO11.10 mice, whereas no antigen-dependent alteration was observed in the frequency of CD8*αα* and CD8*αβ* IELs expressing CD103 (Figure 3(c)). However, the expression of CD103 was significantly augmented in subpopulation of CD8*αα* ^+^ and CD8*αβ* ^+^ IELs when BALB/c mice were fed with OVA, as well as in CD8*αα* ^+^ cells in ip immunized mice. Conversely, in DO11.10 mice, this marker was significantly reduced in CD8*αα* ^+^ cells upon immunization with OVA by ip route (Figure 3(c)).

**Figure 4:**
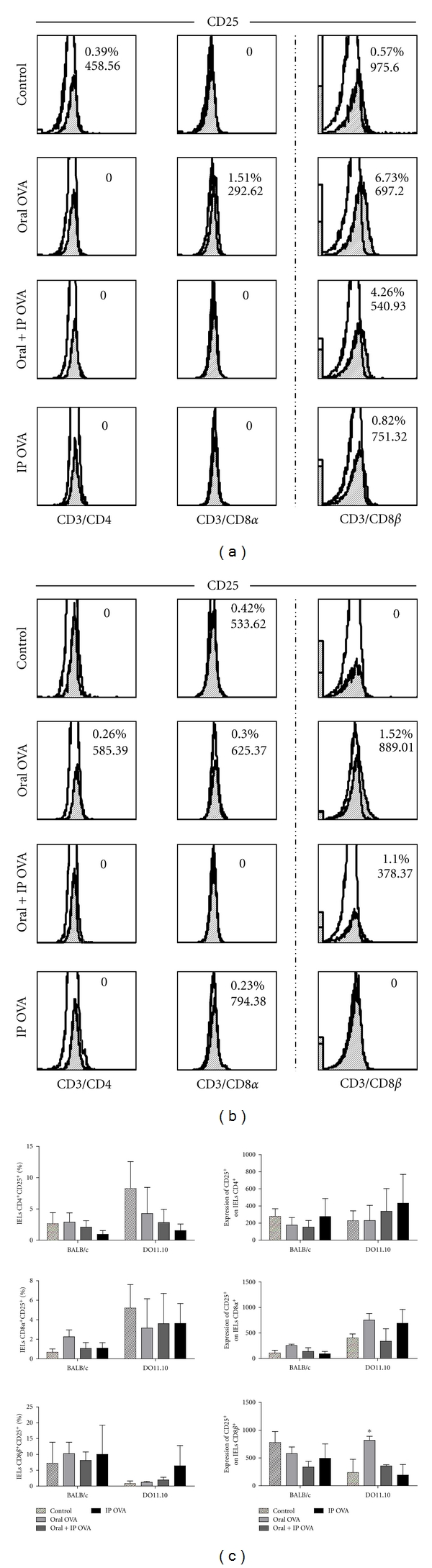
Effects of the treatments with OVA on the frequency of CD25^+^ cells in the small intestine. Freshly isolated IELs from BALB/c (a) and DO11.10 (b) mice were gated for CD3^+^/CD4^+^, CD3^+^/CD8*α* ^+^, and CD3^+^/CD8*β* ^+^ cells and analyzed for expression of CD25 by flow cytometry. Blank histograms indicate isotype control staining. CD3^+^/CD8*β* ^+^ subset illustrated at the right column is part of the CD3^+^/CD8*α* ^+^ population. (c) Data represent mean ± SEM (*N* = 5), in three independent experiments. An increased frequency of CD25^+^ cells can be observed only in CD8*β* subset of IELs from DO11.10 mice treated with OVA by oral route.

Frequency and expression of CD25^+^ cells in subsets of IELs of mice BALB/c and DO11.10 are illustrated in histograms of (Figures 4 (a) and 4(b) resp.). No antigen-dependent alteration was observed in the frequency of IELs expressing this marker in both DO11.10 and BALB/c mice, except in CD8*β* ^+^ subset of IELs in which this marker was upregulated by treatments with OVA by oral route (Figure 4(c)).

### 3.3. Evaluation of Foxp3 Expression in IELs after Administration of OVA

**Figure 5:**
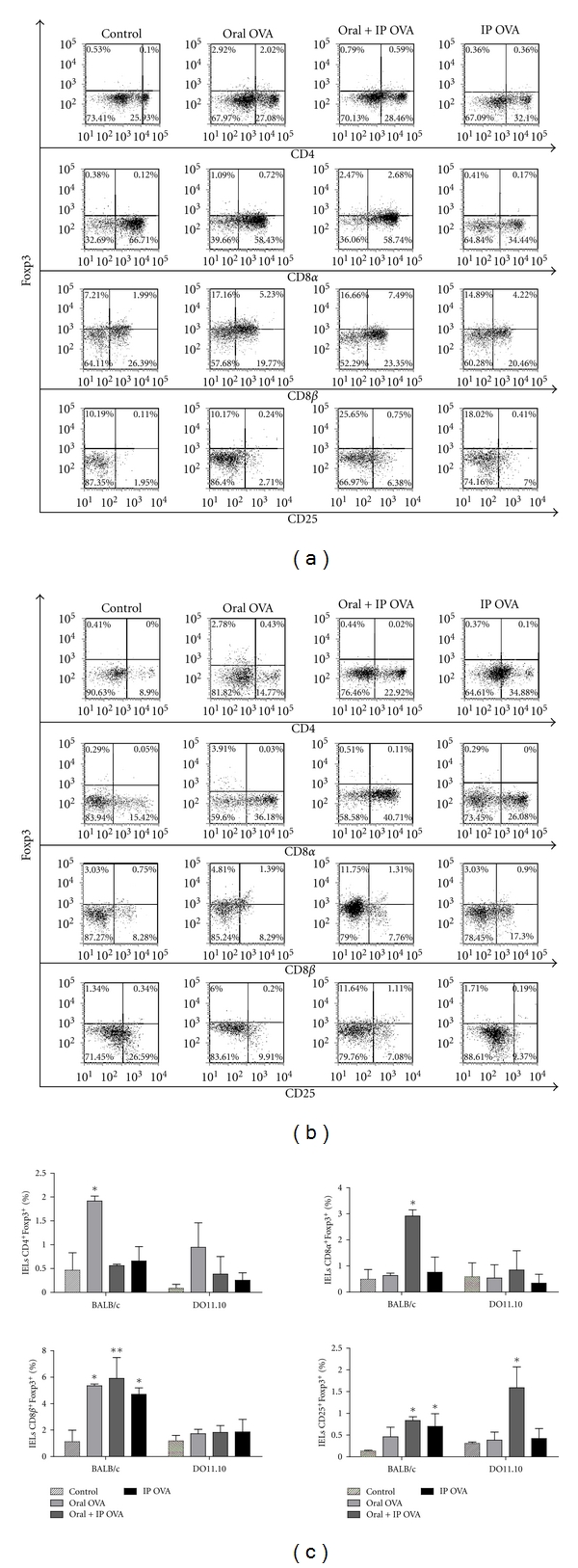
Effects of the treatments with OVA on the frequency of Foxp3^+^ cells in the small intestine. IELs were isolated from BALB/c (a) and DO11.10 (b) mice, and the frequency of Foxp3^+^ cells were analyzed on for CD3^+^/CD4^+^, CD3^+^/CD8*α* ^+^, and CD3^+^/CD8*β* ^+^ cells. (c) Data represent mean ± SEM (*N* = 5), in three independent experiments. Treatments of BALB/c mice with OVA resulted in increase of the frequency of Foxp3^+^ cells in all IEL populations. The oral + ip treatment of DO11.10 mice has increased the frequency of Foxp3^+^cells in IELs CD25^+^.

To assess possible changes in the frequency of regulatory T cells after oral and/or ip administration of OVA, IELs from BALB/c and DO11.10 mice were stained with anti-Foxp3 and analyzed by flow cytometry in CD4^+^, CD8*αα* ^+^, CD8*αβ* ^+^, and CD25^+^ subsets. As shown in Figures 5(a) and 5(c), we observed that oral administration of OVA to BALB/c mice resulted in elevation of the frequency of Foxp3^+^ cells in CD4^+^ and CD8*αβ* ^+^subsets. Following parenteral administration of OVA, the frequency of cells Foxp3^+^ in IELs of BALB/c mice was more elevated in the CD8*αα* ^+^, CD8*αβ* ^+^, and CD25^+^ subsets. On the other hand, only the oral + ip treatment of DO11.10 mice increased the frequency of Foxp3^+^ cells in the CD25^+^ subset (Figures 5(b) and 5(c)).

### 3.4. Effect of Treatments with OVA on Cytokine mRNA Expression in IELs of BALB/c and DO11.10 Mice

**Figure 6:**
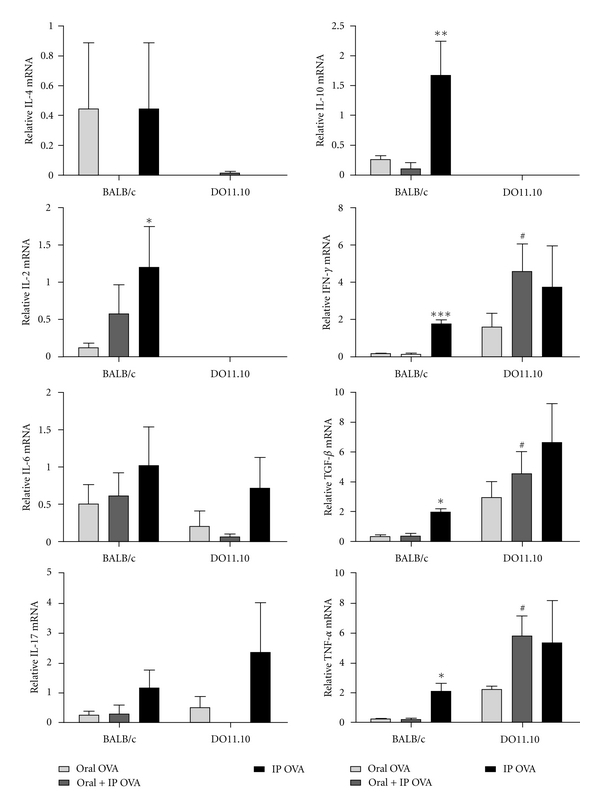
Quantitative mRNA analysis of cytokine expression in intestinal IELs. Total RNA was extracted from freshly isolated IELs of BALB/c and DO11.10 mice (*N* = 5), and cDNA of IL-2, IL-4, IL-6, IL-10, IL-17, IFN-*γ*, TNF-*α*, and TGB-*β* was made using PureLink Micro-to-Midi Total RNA Purification System (Invitrogen, SP, Brazil,). Quantitative PCR analysis was performed with the ABI 7500 Fast Real-Time PCR system (Applied Biosystems), in four replicates, with the TaqMan Mastermix (Applied Biosystems). Samples were normalized to 18S rRNA, and an arbitrary value of 1 was given to control group (naïve mice) for the normalization, and the remaining samples were plotted relative to that value. IELs from BALB/c mice immunized by ip route show an increase in the expression of mRNA of cytokines IL-10, IL-2, IFN-*γ*, TGF-*β*, and TNF-*α*. IELs from DO11.10 mice fed with OVA and challenged by ip route showed increased expression of mRNA for cytokines IFN-*γ*, TGF-*β*, and TNF-*α*. The results are representative of three independent experiments.

In addition to phenotypic analysis, expression of pro-(IL-2, IFN-*γ*, IL-6, IL-17, and TNF-*α*) and anti-inflammatory (IL-10, IL-4, and TGF-*β*) cytokines has been assessed by real-time PCR from extracts of IELs of the small intestine of BALB/c and DO11.10 mice treated with OVA. The results are summarized in Figure 6. It is possible to notice that IELs from OVA-treated mice of both strains present opposite profiles in relation to the gene expression of most cytokines examined. IELs from tolerant BALB/c mice (oral and oral + ip groups) showed a diminished expression of mRNA for cytokines IL-10, IL-2, IFN-*γ*, TGF-*β*, and TNF-*α* in comparison to those from mice immunized by ip route. IELs from DO11.10 mice treated with OVA by oral + ip and ip routes showed an elevated expression of IL-6, IL17, TNF-*α*, and TGF-*β*, although differences were not significant in comparison with the oral group. IELs from DO11.10 mice that received OVA by oral + ip, however, showed a mRNA expression for TNF-*α*, IFN-*γ*, and TGF-*β* significantly more elevated than IELs from BALB/c mice of the same group.

## 4. Discussion

Failure in the induction of oral tolerance seems to be associated with modifications in the gastrointestinal mucosa permeability and, especially, with the immunoregulation that occurs in this environment [19, 20]. Results obtained previously in our laboratory showed that transgenic DO11.10 and BALB/c mice differ in their immune response to oral OVA. While DO11.10 mice develop a specific humoral immune response after the ingestion of native ovalbumin, the BALB/c mice become tolerant to OVA. The transgenic mice fed with ovalbumin produced an immune response that is a mixed of TH1/TH2, with prevalence of a TH1 pattern [12]. In this work, we observed that even before the oral treatment with OVA, the DO11.10 mice have showed morphological modifications in the intestinal epithelium villi and of the muscular layer in the intestinal tissue. Our results showed a deepening of changes in the epithelium of small intestine in DO11.10 mice treated with OVA, which are consistent to an inflammatory process. These changes have not been observed in the intestinal epithelium of BALB/c mice, which have been presented a good preservation of the villi. A chronic inflammatory process, with lymphocytic infiltration in the lamina propria and increased number of IELs in the epithelium has been shown in double-transgenic mice that express the haemagglutinin from influenza virus A (HA) and TCR HA-specific. The inflammatory reaction, however, is kept under control by the generation of regulatory T cells [14]. 

Deletion of self-reactive lymphocytes constitutes one of main mechanisms of peripheral tolerance induction and probably of oral tolerance induction [21, 22]. Conversely, intestinal inflammation has been correlated with failure in the induction of apoptosis in lymphocytes present in the mucosa [23]. The initial hypothesis of our study was that resistance to the induction of oral tolerance in DO11.10 might be due to failure in the deletion of OVA-specific lymphocytes in the intestinal mucosa after ingestion of the antigen. Indeed, treatment with OVA by oral and/or ip routes resulted in reduction of IELs in the DO11.10 mice. Besides that, a remarkable reduction in OVA-specific cells (KJ1-26) was observed in small intestine of DO11.10 mice treated with OVA. Previous work has shown that the administration of OVA results in marked reduction of mature lymphocytes KJ1-26^+^ in the blood and peripheral lymphoid organs [16]. However, a substantial portion of the IELs and lymphocytes from LP from the DO11.10 mice carries a second nonclonotypical TCR, probably due to the incomplete allelic exclusion of the endogenous TCR*α* during the rearrangement process in the thymus [21, 24]. Part of the alternative TCR seems to be specific to antigens from the intestinal environment as DO11.10/SCID or DO11.10/RAG2^−^/^−^ mice do not exhibit reactivity to antigens from the intestinal microbiota [25, 26]. Thus, a possible explanation for the escape of DO11.10 from oral tolerance in spite of the occurrence of TCR OVA-specific cells deletion would be the activation of T cells bearing alternative TCR, that would result in local inflammatory response, with activation of nontransgenic IELs carrying TCR to other than OVA epitopes. 

The IELs have phenotype similar to either effector or effector memory of cells that are present in other peripheral lymphoid organs [27], presenting a high expression of the CD103 marker (*α*_E_B_7_), an *α*-integrin responsible for the retention of IELs in the intestinal epithelium [28]. This molecule has been associated to the immuneregulation in the mucosa as it is expressed by regulatory T lymphocytes [29, 30] as well as by dendritic cells involved in the generation of regulatory T cells [31, 32]. IELs from small intestine of the BALB/c mice treated with OVA orally and/or parenteral have shown marked increase in the expression of CD103, in all populations studied: TCD4, TCD8*α*, and TCD8*β*. Furthermore, IELs from orally treated DO11.10 mice have shown no changes in the expression of CD103 in any cell subset. In contrast, TGN immunized intraperitoneally with OVA showed an accentuated reduction in the expression of CD103 in these cells.

Several populations of regulatory cells have been described in oral tolerance, including IL-10 producer cells termed Tr1, TGF-*β* producing cells called Th3 and TCD4^+^/CD25^+^, and its relative importance in the establishment of oral tolerance is still under investigation [1, 20]. Some of these studies have emphasized the role of the CD8*αα* IELs in the immunoregulation that occurs in the intestinal mucosa [33, 34]. In this work, we did not observe changes in the frequency of this subset of IELs in either BALB/c or DO11.10 mice immunized orally or parenterally with OVA.

There is a consensus in the literature that the oral tolerance is related to the induction of antigen-specific regulatory T cells either by direct or cross-presentation of antigens from the enterocytes [20, 21, 34]. Our results have shown that the ingestion of OVA led to an increase in the frequency of TCD4^+^/Foxp3^+^, TCD8*α* ^+^/Foxp3^+^, TCD8*β* ^+^/Foxp3^+^, and TCD25^+^/Foxp3^+^ amongst the IELs of BALB/c mice, thus indicating that the establishment of tolerance in wild-type mouse are associated with the increase of regulatory T cells. The frequency of TCD25^+^/Foxp3^+^ cells has also increased in the IELs from TGN treated with OVA by oral + ip routes. However, this increase has not been sufficient to inhibit the inflammation that has settled in the small intestinal mucosa of the TGN after treatment with the protein.

TCD8^+^ cells also play an important role in the homeostatic maintenance of the intestinal epithelium. In this regard, TCD8*αβ* suppressor cells have been correlated with the establishment of antigen specific oral tolerance [30, 35, 36]. Although the ingestion of OVA leads to an increase in the frequency of Foxp3^+^ cells in all populations of IEL from both BALB/c and DO11.10 mice, our results showed that it is in the TCD8*β* ^+^ population of IELs that occurs the highest frequency of cells carrying this suppression marker.

Due to the exposure of mucosal epithelium to a huge amount of strange antigens, the cytokines IFN-*γ* and interleukin-(IL-)4 are produced spontaneously under physiological conditions by IELs [37]. In our study, we observed that the expression of IL-2, IL-10, IFN-*γ*, TGF-*β*, and TNF-*α* mRNA was smaller in IELS from BALB/c mice fed OVA and then challenged by ip route than in those that received OVA only by ip route; the levels of IL-17 and IL-6 mRNA were also reduced, but not significantly, in IELs from BALB/c mice. Instead, IELS from transgenic mice of the oral + ip group showed levels of IL-6, IFN-*γ*, TGF-*β*, and TNF-*α* mRNA similar to or higher than the animals immunized only by ip route.

The cytokines IL-10 and TGF-*β* were always related to anti-inflammatory reactions [1, 20]. More recently, however, these cytokines have been associated with inflammatory reactions by activating the process of differentiation of Th17 cells [38, 39]. TCD4^+^ cells that secrete IL-17, Th17 cells, are pathogenic in autoimmune diseases, and their development and expansion is driven by the cytokines IL-6, TGF-beta, IL-21, IL-1, and IL-23 [39, 40]. Recent studies have revealed a considerable number of IL-17-producing cells amongst the TCD4^+^ cells in the intestinal mucosa [41]. Although these cells are important to establish a protective immune response against intestinal bacteria, they can also be responsible for inducing inflammatory response and the development of immunological disorders in the presence of IL-6 and/or IL-23 at mucosal sites [41, 42]. Despite the natural occurrence of higher expression of IL-6, TGF-*β*, and TNF-*α* mRNA in the DO11.10 IELS than in those of BALB/c mice, there was no significant change in IL-17 mRNA after administration of OVA orally and/or ip in both BALB/c and DO11.10 mice.

Th1-mediated immune responses are considered to be the primary mediators of most autoimmune and chronic inflammatory diseases. Th17, however, has emerged as a key protagonist in a number of inflammatory diseases. It has been shown that CD8^+^ Treg cells can suppress both Th1 and Th17 responses, being capable of mediating oral tolerance to OVA independently of their CD4^+^ counterparts in a normal immune system [43]. In the present work, the increase in the frequency of CD8*αβ* ^+^Foxp3^+^ cells among IELS of BALB/c mice after ingestion of OVA could explain the absence or reduced expression of IL-6, TGF-*β*, TNF-*α*, and IFN-*γ*.

Recent studies have shown that inflammatory bowel diseases (IBD) such as ulcerative colitis and Crohn's disease may be related to the loss of tolerance to self-antigens or normal flora [44]. Our results indicate that the structural disorder observed in epithelium of intestinal villi of transgenic mice would be a consequence of the preferential expression of pro-inflammatory cytokines by their IELs, in the absence of an efficient immunoregulation. This inflammatory state in intestinal environment may contribute to the impairment of oral tolerance to OVA in DO11.10 mice.

## 5. Conclusion

Taken together, the results of this study indicate that exposure to OVA orally causes IELs of the small intestine of TGN mice assume inflammatory characteristics, whereas in BALB/c antigen intake leads to the development of IELs with characteristics of regulatory cells. Thus, we speculate that the establishment of oral tolerance in transgenic mice is severely impaired by changes in the amounts and arrangements of T cells during the development of intestinal tissues that compromise the cellular interactions involved in the processes of mucosal immunity.

## Supplementary Material

The effects of administration of OVA on the frequency of IELS expressing TCR *α*:*β* and TCR *γ*:*δ*, CD103, CD25, CTLA-4 and Foxp3 in CD4^+^, CD8*α* ^+^ and CD8*β* ^+^ T-cell subsets was additionally evaluated in IELs from small intestines of BALB/c and DO11.10 mice by flow cytometry. The results are summarized in Supplemental Figures 1 and 2.

## Figures and Tables

**Table 1:** Number of cells recovered from 40/70%-Percoll interface

	Cells from^ (a,b)^			
	BALB/C		DO11.10	
Treatments				
Control	7.07	±2.05	4.27	±1.51
Oral ova	6.50	±1.91	1.57*	±0.72
Oral + IP ova	11.62	±4.97	1.00*	±0.29
IP ova	12.75	±3.41	0.55**	±0.08

^(a)^Number of cells represents the mean ± SEM × 10^7^ cells; ^(b)^Data were obtained from 3-4 independent experiments; *P < 0.05; **P < 0.01.

## Abbreviations

GALT: Gut-associated lymphoid tissue
IBD: Inflammatory bowel disease
IELs: Intestinal epithelial cells
IFN-*γ*: Interferon-*γ*
LP: Lamina propria
MLN: Mesenteric lymph node
OVA: Ovalbumin
PP: Peyer's patch
TGF-*β*: Transforming growth factor-*β*
TCR: T-cell receptor
TGN: Transgenic mice
mAb: Monoclonal antibody.

## References

[1] Dubois B, Goubier A, Joubert G, Kaiserlian D (2005). Oral tolerance and regulation of mucosal immunity. *Cellular and Molecular Life Sciences*.

[2] Friedman A, Weiner HL (1994). Induction of anergy or active suppression following oral tolerance is determined by antigen dosage. *Proceedings of the National Academy of Sciences of the United States of America*.

[3] Chen Y, Inobe JI, Marks R, Gonnella P, Kuchroo VK, Weiner HL (1995). Peripheral deletion of antigen-reactive T cells in oral tolerance. *Nature*.

[4] Lee H-O, Miller SD, Hurst SD, Tan LJ, Cooper CJ, Barrett TA (2000). Interferon gamma induction during oral tolerance reduces T-cell migration to sites of inflammation. *Gastroenterology*.

[5] Parameswaran N, Samuvel DJ, Kumar R (2004). Oral tolerance in T cells is accompanied by induction of effector function in lymphoid organs after systemic immunization. *Infection and Immunity*.

[6] Mowat AM (2003). Anatomical basis of tolerance and immunity to intestinal antigens. *Nature Reviews Immunology*.

[7] Toussirot EA (2002). Oral tolerance in the treatment of rheumatoid arthritis. *Current Drug Targets—Inflammation & Allergy*.

[8] Kunisawa J, Takahashi I, Kiyono H (2007). Intraepithelial lymphocytes: their shared and divergent immunological behaviors in the small and large intestine. *Immunological Reviews*.

[9] Hayday A, Theodoridis E, Ramsburg E, Shires J (2001). Intraepithelial lymphocytes: exploring the third way in immunology. *Nature Immunology*.

[10] Lefrancois L (1991). Phenotypic complexity of intraepithelial lymphocytes of the small intestine. *The Journal of Immunology*.

[11] Wang HC, Zhou Q, Dragoo J, Klein JR (2002). Most murine CD8+ intestinal intraepithelial lymphocytes are partially but not fully activated T cells. *The Journal of Immunology*.

[12] Simioni P, Fernandes LGR, Gabriel DL, Tamashiro WMSC (2004). Induction of systemic tolerance in normal but not in transgenic mice through continuous feeding of ovalbumin. *Scandinavian Journal of Immunology*.

[13] Tamauchi H, Yoshida Y, Sato T (2005). Oral antigen induces antigen-specific activation of intraepithelial CD4+ lymphocytes but suppresses their activation in spleen. *Immunobiology*.

[14] Westendorf AM, Templin M, Geffers R (2005). CD4+ T cell mediated intestinal immunity: chronic inflammation versus immune regulation. *Gut*.

[15] Goto M, Hachimura S, Ametani A (2003). Antigen feeding increases frequency and antigen-specific proliferation ability of intraepithelial TCD4+ T cells in a *αβ* T cell receptor transgenic mice. *Bioscience, Biotechnology, and Biochemistry*.

[16] Murphy KM, Heimberger AB, Loh DY (1990). Induction by antigen of intrathymic apoptosis of CD4^+^CD8^+^TCR^lo^ thymocytes in vivo. *Science*.

[17] Montufar-Solis D, Klein JR (2006). An improved method for isolating intraepithelial lymphocytes (IELs) from the murine small intestine with consistently high purity. *Journal of Immunological Methods*.

[18] Livak KJ, Schmittgen TD (2001). Analysis of relative gene expression data using real-time quantitative PCR and the 2-ΔΔCT method. *Methods*.

[19] Kraus TA, Toy L, Chan L, Childs J, Mayer L (2004). Failure to induce oral tolerance to a soluble protein in patients with inflammatory bowel disease. *Gastroenterology*.

[20] Saurer L, Mueller C (2009). T cell-mediated immunoregulation in the gastrointestinal tract. *Allergy*.

[21] Liu Z, Lefrançois L (2004). Intestinal epithelial antigen induces mucosal CD8 T cell tolerance, activation, and inflammatory response. *The Journal of Immunology*.

[22] Magnusson FC, Liblau RS, Von Boehmer H (2008). Direct presentation of antigen by lymph node stromal cells protects against CD8 T-cell-mediated intestinal autoimmunity. *Gastroenterology*.

[23] Di Sabatino A, Ciccocioppo R, D’Alò S (2001). Intraepithelial and lamina propria lymphocytes show distinct patterns of apoptosis whereas both populations are active in fas based cytotoxicity in coeliac disease. *Gut*.

[24] Hurst SD, Sitterding SM, Ji S, Barrett TA (1997). Functional differentiation of T cells in the intestine of T cell receptor transgenic mice. *Proceedings of the National Academy of Sciences of the United States of America*.

[25] Saparov A, Kraus LA, Cong Y (1999). Memory/effector T cells in TCR transgenic mice develop via recognition of enteric antigens by a second, endogenous TCR. *International Immunology*.

[26] Zhou P, Borojevic R, Streutker C, Snider D, Liang H, Croitoru K (2004). Expression of dual TCR on do11.10 T cells allows for ovalbumin-induced oral tolerance to prevent T cell-mediated colitis directed against unrelated enteric bacterial antigens. *The Journal of Immunology*.

[27] Cheroutre H, Madakamutil L (2004). Acquired and natural memory T cells join forces at the mucosal front line. *Nature Reviews Immunology*.

[28] Parker CM, Cepek KL, Russell GJ (1992). A family of *β*7 integrins on human mucosal lymphocytes. *Proceedings of the National Academy of Sciences of the United States of America*.

[29] Lehmann J, Huehn J, De la Rosa M (2002). Expression of the integrin *α*
_*E*_
*β*
_7_ identifies unique subsets of CD25^+^ as well as CD25^−^ regulatory T cells. *Proceedings of the National Academy of Sciences of the United States of America*.

[30] Ho J, Kurtz CC, Naganuma M, Ernst PB, Cominelli F, Rivera-Nieves J (2008). A CD8^+^/CD103^high^ T cell subset regulates TNF-mediated chronic murine ileitis. *The Journal of Immunology*.

[31] Annacker O, Coombes JL, Malmstrom V (2005). Essential role for CD103 in the T cell-mediated regulation of experimental colitis. *The Journal of Experimental Medicine*.

[32] Siddiqui KR, Powrie F (2008). CD103+ GALT DCs promote Foxp3+ regulatory T cells. *Mucosal Immunology*.

[33] Poussier P, Ning T, Banerjee D, Julius M (2002). A unique subset of self-specific intraintestinal T cells maintains gut integrity. *Journal of Experimental Medicine*.

[34] Rimoldi M, Chieppa M, Salucci V (2005). Intestinal immune homeostasis is regulated by the crosstalk between epithelial cells and dendritic cells. *Nature Immunology*.

[35] Feinberg MB, Silvestri G (2002). TS cells and immune tolerance induction: a regulatory renaissance?. *Nature Immunology*.

[36] Westendorf AM, Fleissner D, Deppenmeier S (2006). Autoimmune-mediated intestinal inflammation-impact and regulation of antigen-specific CD8+ T Cells. *Gastroenterology*.

[37] Carol M, Lambrechts A, Van Gossum A, Libin M, Goldman M, Mascart-Lemone F (1998). Spontaneous secretion of interferon *γ* and interleukin 4 by human intraepithelial and lamina propria gut lymphocytes. *Gut*.

[38] Li MO, Wan YY, Flavell RA (2007). T cell-produced transforming growth factor-*β*1 controls T cell tolerance and regulates Th1- and Th17-cell differentiation. *Immunity*.

[39] Wynn TA (2005). TH-17: a giant step from TH1 and TH2. *Nature Immunology*.

[40] Sutton CE, Lalor SJ, Sweeney CM, Brereton CF, Lavelle EC, Mills KHG (2009). Interleukin-1 and IL-23 induce innate IL-17 production from *γ*Δ T cells, amplifying Th17 responses and autoimmunity. *Immunity*.

[41] Honda K, Takeda K (2009). Regulatory mechanisms of immune responses to intestinal bacteria. *Mucosal Immunology*.

[42] Kitani A, Xu L (2008). Regulatory T cells and the induction of IL-17. *Mucosal Immunology*.

[43] Arnaboldi PM, Roth-Walter F, Mayer L (2009). Suppression of Th1 and Th17, but not Th2, responses in a CD8+ T cell-mediated model of oral tolerance. *Mucosal Immunology*.

[44] Boirivant M, Amendola A, Butera A (2008). Intestinal microflora and immunoregulation. *Mucosal Immunology*.

