# Erratum to “Phenotypical and Functional Analysis of Intraepithelial Lymphocytes from Small Intestine of Mice in Oral Tolerance”

**DOI:** 10.1155/2012/620278

**Published:** 2012-05-22

**Authors:** Maristela Ruberti, Luis Gustavo Romani Fernandes, Patricia Ucelli Simioni, Dirce Lima Gabriel, Áureo Tatsumi Yamada, Wirla Maria da Silva Cunha Tamashiro

**Affiliations:** ^1^Department of Genetics, Evolution and Bioagents, Institute of Biology, University of Campinas (UNICAMP), CP 6109, 13083-970 Campinas, SP, Brazil; ^2^Department of Histology and Embryology, Institute of Biology, University of Campinas (UNICAMP), CP 6109, 13083-970 Campinas, SP, Brazil

In this work, we evaluated the effects of administration of OVA on phenotype and function of intraepithelial lymphocytes (IELs) from small intestine of transgenic (TGN) DO11.10 and wild-type BALB/c mice. While the small intestines from BALB/c presented a well-preserved structure, those from TGN showed an inflamed aspect. The ingestion of OVA induced a reduction in the number of IELs in small intestines of TGN, but it did not change the frequencies of CD8+ and CD4+ T-cell subsets. Administration of OVA via oral + i.p. increased the frequency of CD103+ cells in CD4+ T-cell subset in IELs of both BALB/c and TGN mice and elevated its expression in CD8a+ and CD8*β*+ T-cell subsets in IELs of BALB/c mice. The frequency of Foxp3+ cells increased in all subsets in IELs of BALB/c treated with OVA; in IELs of TGN, it increased only in CD25+ subset. IELs from BALB/c tolerant mice had lower expression of all cytokines studied, whereas those from TGN showed high expression of inflammatory cytokines, especially of IFN-*γ*, TGF-*β*, and TNF-*α*. Overall, our results suggest that the inability of TGN to become tolerant may be related to disorganization and altered proportions of inflammatory/regulatory T cells in its intestinal mucosa.

