# Supplementary figures and images for: Phenotypical and Functional Analysis of Intraepithelial Lymphocytes from Small Intestine of Mice in Oral Tolerance
Source: Clin Dev Immunol. 2012 Feb 7;2012:208054. doi: 10.1155/2012/208054 (PMC3287057; doi:10.1155/2012/208054)

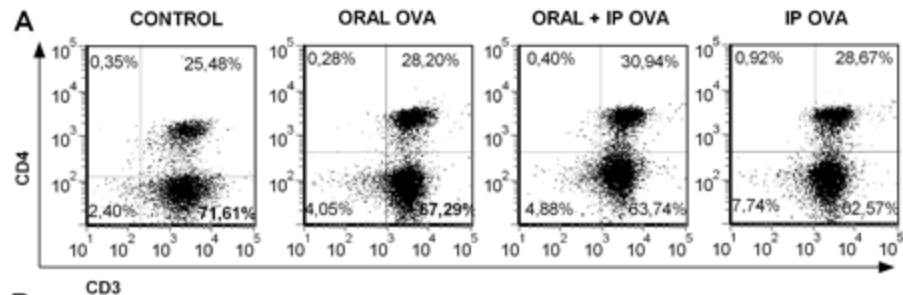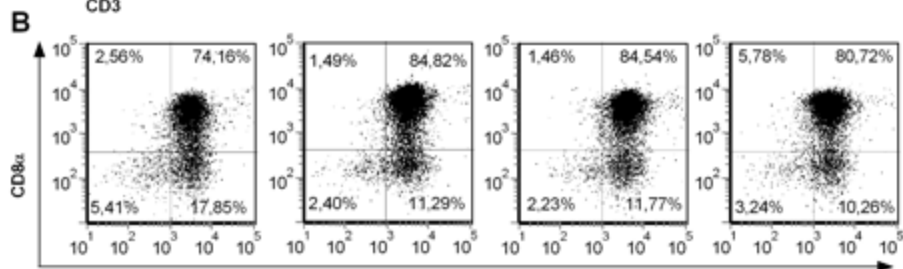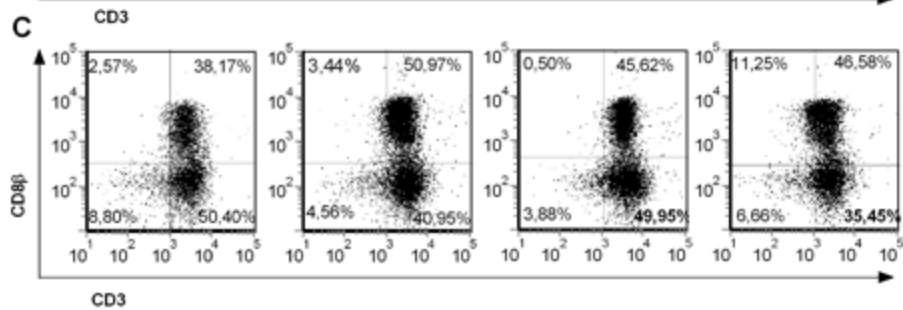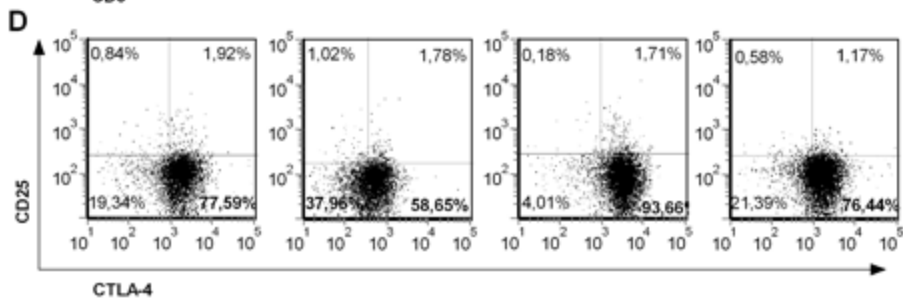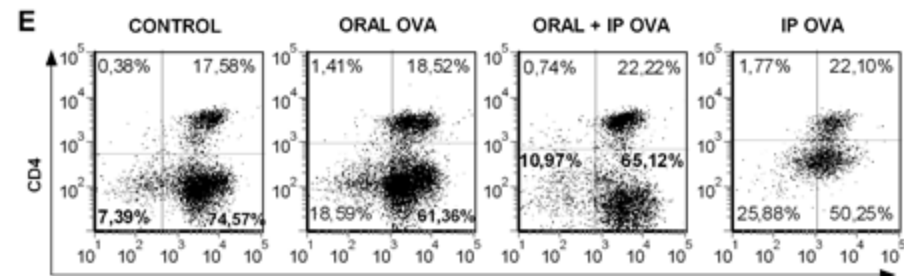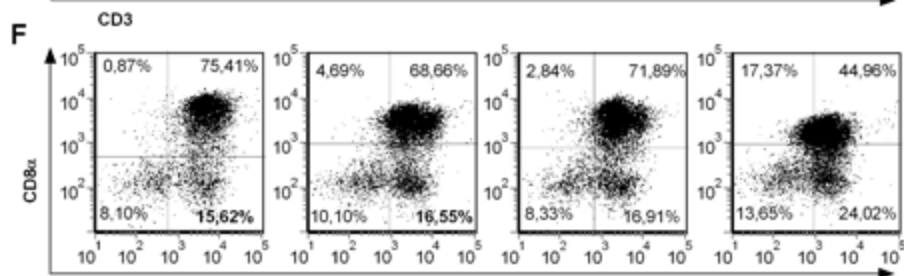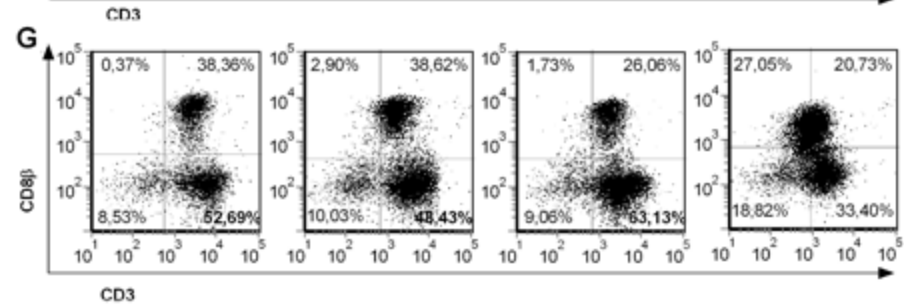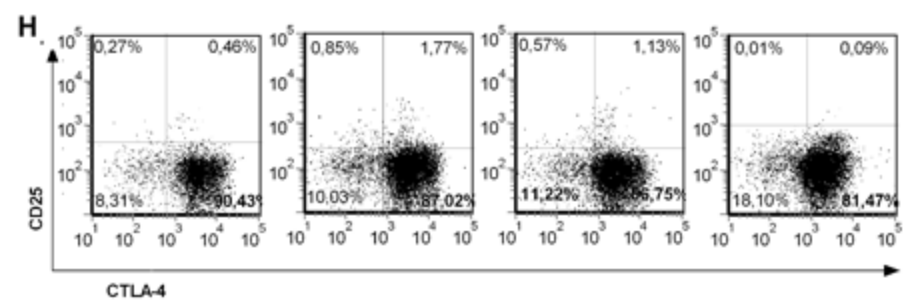

Supplement: Supplementary file 1 — The effects of administration of OVA on the frequency of IELS expressing TCR α:β and TCR γ:δ, CD103, CD25, CTLA-4 and Foxp3 in CD4+, CD8α + and CD8β + T-cell subsets was additionally evaluated in IELs from small intestines of BALB/c and DO11.10 mice by flow cytometry. The results are summarized in Supplemental Figures 1 and 2. [file 208054.f1.pdf]

**A**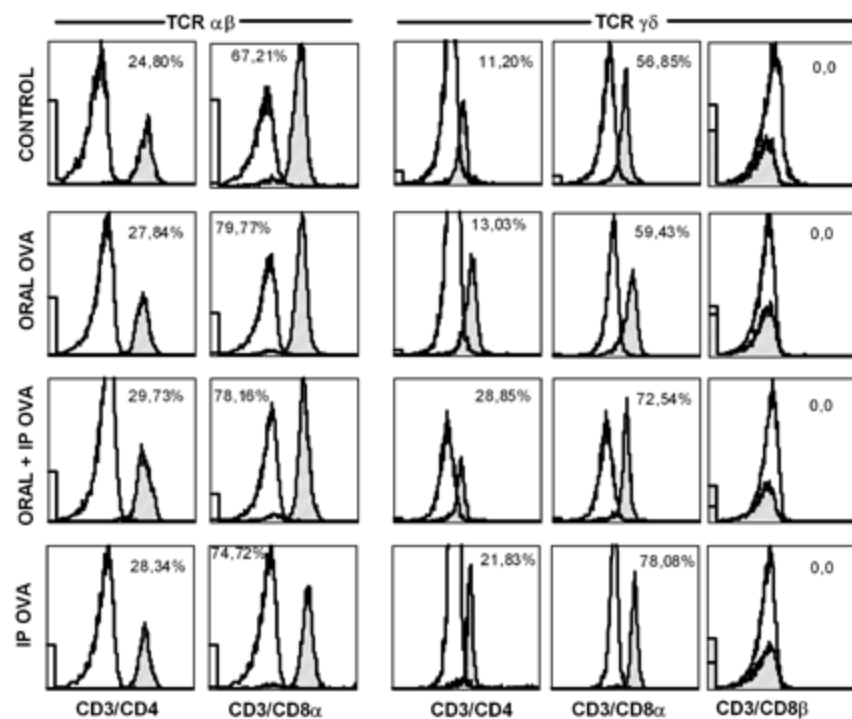**B**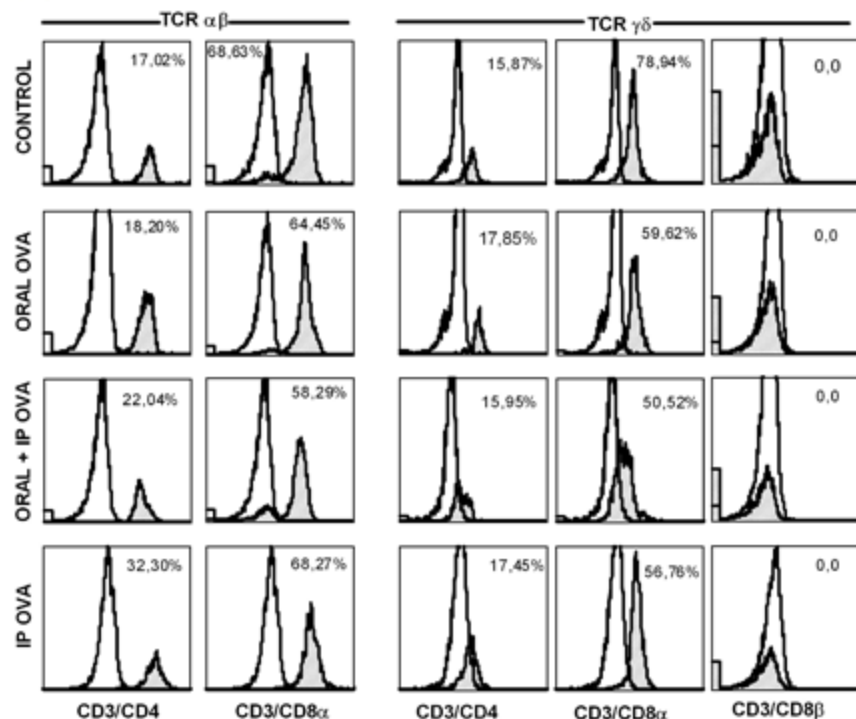**C**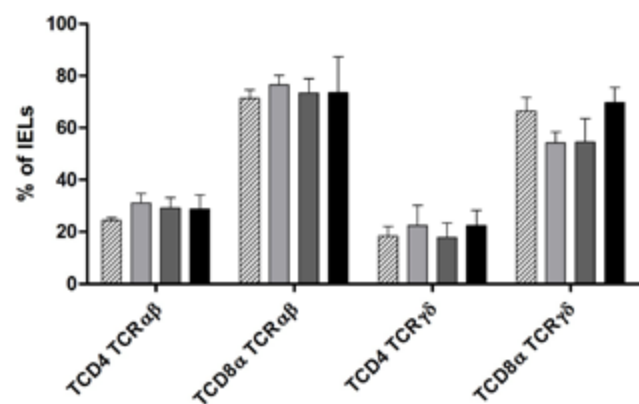**D**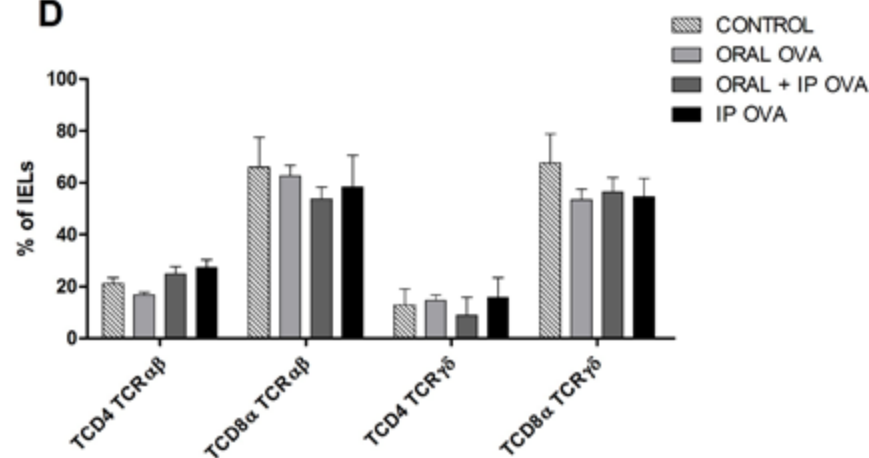

Supplement: Supplementary file 2 [file 208054.f2.pdf]
